# Functional Expansion of the Skin Microbiome: A Pantothenate-Producing *Rothia* Strain Confers Anti-Inflammatory and Photoaging-Protective Effects

**DOI:** 10.3390/ijms262412058

**Published:** 2025-12-15

**Authors:** Hye-Been Kim, Gihyeon Kim, Eunjin Park, Hyeyoun Kim, Byung Sun Yu, Dong-Geol Lee, Chun Ho Park, HyungWoo Jo, Hansoo Park

**Affiliations:** 1 Research and Innovation Center, Cosmax BTI, Seongnam 13486, Republic of Korea; 2 Genome and Company, Suwon 16229, Republic of Korea; 3 Department of Biomedical Sciences, College of Bio-Convergence, Dankook University, Cheonan 31116, Republic of Korea; 4 Department of Biomedical Science and Engineering, Gwangju Institute of Science and Technology (GIST), Gwangju 61005, Republic of Korea

**Keywords:** *Rothia kristinae*, skin microbiome, pantothenic acid, anti-inflammatory, comparative genomics, postbiotic

## Abstract

The functional landscape of the skin microbiome is largely defined by dominant genera such as *Cutibacterium* and *Staphylococcus*, whereas rare commensals remain poorly understood. In this study, we identified *Rothia kristinae* BF00107, a skin-resident strain with a complete pantothenate biosynthesis pathway, as a novel postbiotic candidate with distinct dermatological benefits. BF00107 fermentation filtrate suppressed pro-inflammatory cytokines (IL-1β and TNF-α) in keratinocytes and restored extracellular matrix homeostasis in UVB-irradiated fibroblasts by upregulating COL1A1 expression and reducing MMP-1 levels. Consistent with the observed phenotypes, transcriptomic profiling revealed a strain-specific signature characterized by downregulation and upregulation of the expression of inflammatory mediators and barrier- and ECM-associated genes, respectively. Comparative genomics and metabolite profiling confirmed BF00107 as a unique high-pantothenate producer. Supplementation with pantothenic acid reproduced the anti-inflammatory and barrier-supporting effects of the strain, confirming its role as a key effector metabolite. Furthermore, BF00107 passed standard safety assessments, including the Human Repeat Insult Patch Test (HRIPT), Ames, and irritation tests, supporting its suitability for human applications. These findings establish the pantothenate-producing *R. kristinae* BF00107 as the first functionally validated *Rothia* strain with anti-inflammatory and photoaging-protective properties. This study expands the functional scope of the skin microbiome and highlights rare commensals as valuable reservoirs for safe, strain-specific postbiotic development.

## 1. Introduction

The human skin microbiome is integral to barrier function, immune regulation, and aging; however, its functional diversity remains incompletely defined [1,2,3]. The current understanding is dominated by studies of abundant taxa, such as *Cutibacterium*, *Staphylococcus*, and *Corynebacterium*, whereas the roles of less prevalent commensals remain largely unknown. Broadening the taxonomic scope of skin microbiome research is essential for uncovering novel mechanisms by which resident microbes contribute to skin homeostasis. Recent studies have highlighted the functional relevance of underexplored commensals in other host niches, underscoring the need to investigate rare taxa within the skin microbiome [4].

*Rothia*, a genus frequently detected on the human skin, exemplifies this knowledge gap [5,6,7]. Large-scale population studies have reported correlations between *Rothia* abundance and skin phenotypes associated with aging, including reduced tone, elasticity, and hydration. Despite its consistent presence in diverse individuals, the potential contributions of *Rothia* to skin physiology have not been functionally investigated.

A possible mechanistic link is vitamin-associated metabolism. Pantothenic acid (vitamin B5) is a central cofactor for coenzyme A and lipid metabolism. It plays a critical role in keratinocyte differentiation, ceramide biosynthesis, and barrier repair [8,9,10,11,12]. In addition, it modulates inflammatory responses and promotes wound healing. Although pantothenate is classically considered a host- or diet-derived factor, whether skin-resident microbes contribute to its availability or function in the cutaneous environment remains unclear.

Simultaneously, the concept of postbiotics, non-viable microbial preparations, or metabolites with beneficial effects is a promising avenue for microbiome-based interventions. Postbiotics offer safety and stability; however, most studies have focused on well-established genera such as *Lactobacillus*, leaving fewer dominant commensals unexplored as potential bioactive producers.

In this study, we hypothesized that *Rothia kristinae*, a skin-resident commensal, possesses an unrecognized functional potential mediated by metabolite production. To test this hypothesis, we isolated *R. kristinae* BF00107 from human facial skin and conducted a comprehensive evaluation using a combination of comparative genomics, metabolite quantification, transcriptomics, and in vitro functional assays. This study provides the first functional evidence that a *Rothia* strain contributes to skin health by revealing its role in the modulation of inflammation, extracellular matrix homeostasis, and barrier function.

## 2. Results

### 2.1. Cell Viability and Safety Assessments of R. kristinae

**Figure 1:**
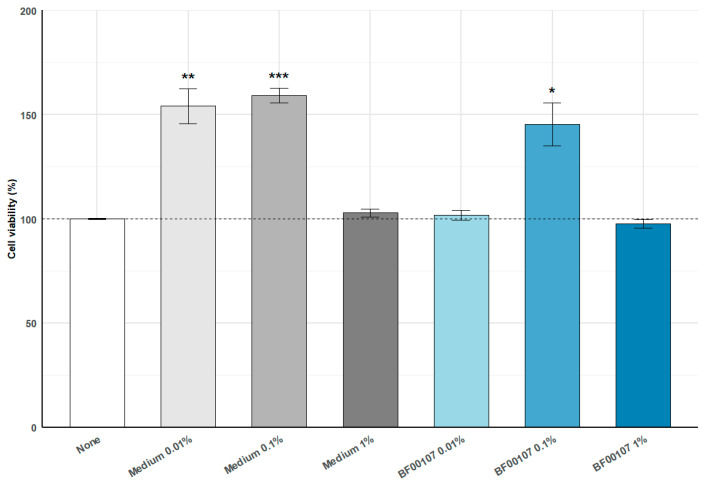
Effects of *R. kristinae* BF00107 on cell viability. Cells were treated with medium (0.01–1%) or *R. kristinae* BF00107 (0.01–1%) for 24 h. Medium 0.01% and 0.1% significantly increased cell viability compared with the none, whereas medium 1% showed no change. BF00107 at 0.1% also enhanced viability, while 0.01% and 1% showed no significant effects. Data are shown as mean ± SD (*n* = 4); statistical significance was assessed using a two-tailed Student’s *t*-test. * *p* < 0.05 vs. None, ** *p* < 0.01 vs. None, *** *p* < 0.001 vs. None.

To evaluate the cell viability an MTT assay was performed following treatment with medium or *R. kristinae* BF00107 at various concentrations (Figure 1). Medium-treated groups showed a significant increase in cell viability at 0.01% (154.02 ± 16.73%, *p* = 0.0050) and 0.1% (159.20 ± 6.84%, *p* = 0.00012) compared with the untreated control, whereas the 1% medium condition did not induce a meaningful change (102.85 ± 3.91%, *p* = 0.279). BF00107 treatment at 0.1% also resulted in a significant elevation in viability (145.27 ± 20.56%, *p* = 0.0189), while the 0.01% and 1% BF00107 groups showed no significant differences from the control (101.79 ± 4.52%, *p* = 0.534; 97.71 ± 4.12%, *p* = 0.394, respectively). These results indicate that low concentrations of medium or BF00107 enhance cell viability without inducing cytotoxicity.

The Human Repeat Insult Patch Test (HRIPT) confirmed that the serum containing *R. kristinae* BF00107 is non-irritant and non-sensitizing, indicating its suitability for cosmetic use. Among 52 subjects tested, no signs of skin irritation were observed during the induction phase, and no sensitization reactions were detected during the challenge phase. These results demonstrate that *R. kristinae* BF00107 is safe with respect to sensitization potential on human skin.

Additionally, three in vitro toxicity assessments were performed. In the reconstructed human epidermis model, *R. kristinae* BF00107 was classified as Non-Irritant (NI) according to the UN GHS “No Category” criteria for skin irritation. Similarly, in the reconstructed human cornea-like epithelium (RhCE) model, the test item was also classified as Non-Irritant (NI), confirming the absence of ocular irritation potential. Furthermore, the bacterial reverse mutation (Ames) test showed no evidence of mutagenic activity under the conditions of this study. Collectively, these findings indicate that *R. kristinae* BF00107 is non-irritant, non-sensitizing, and non-mutagenic, supporting its overall safety for topical applications.

### 2.2. Anti-Inflammatory Effect of R. kristinae

**Figure 2:**
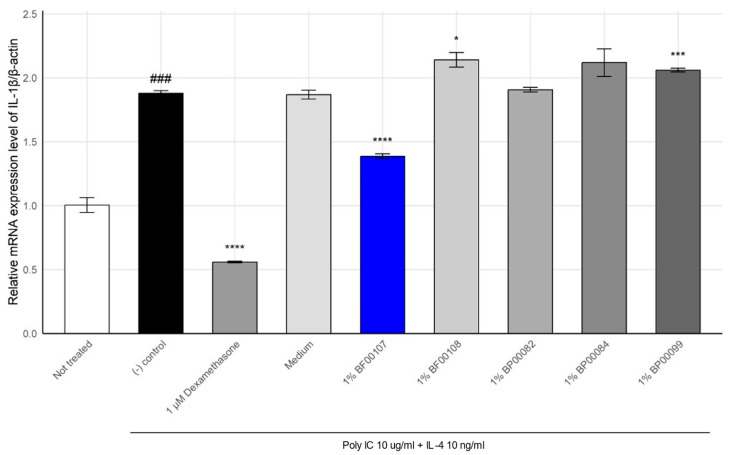
Effect of *Rothia* species on the mRNA expression levels of IL-1β in HaCaT cells. ### *p* < 0.001 vs. None, * *p* < 0.05 vs. Inducer, *** *p* < 0.001 vs. Inducer, **** *p* < 0.0001 vs. Inducer.

To evaluate the anti-inflammatory properties of *R. kristinae* BF00107, we quantified the expression of IL-1β, a key pro-inflammatory cytokine, in keratinocytes stimulated with poly I:C and IL-4 (Figure 2). We further compared its efficacy with that of other fermentation filtrates derived from different *Rothia* species (BF00108, BP00082, BP00084, and BP00099). Inflammatory stimulation significantly increased IL-1β expression to 1.878 ± 0.0215 compared to that in the untreated group (1.003 ± 0.0584, *p* = 1.48 × 10^−4^), confirming the successful induction of inflammation. Treatment with 1 μM dexamethasone, a known anti-inflammatory agent, markedly suppressed IL-1β expression to 0.558 ± 0.0067 (*p* = 5.05 × 10^−7^).

Among the fermentation filtrates from the *Rothia* genus, BF00107 showed the greatest reduction in IL-1β expression (1.384 ± 0.0200, *p* = 7.32 × 10^−5^ vs. control), demonstrating a notable anti-inflammatory effect. In contrast, filtrates from other *Rothia* species failed to suppress IL-1β effectively. In some cases, these filtrates further elevated its expression levels (e.g., BF00108: 2.139 ± 0.0573, BP00099: 2.060 ± 0.0142).

These results suggest that anti-inflammatory activity within the *Rothia* genus is species-specific, with *R. kristinae* BF00107 exerting a distinctly superior effect on the downregulation of IL-1β expression compared to other *Rothia* strains.

**Figure 3:**
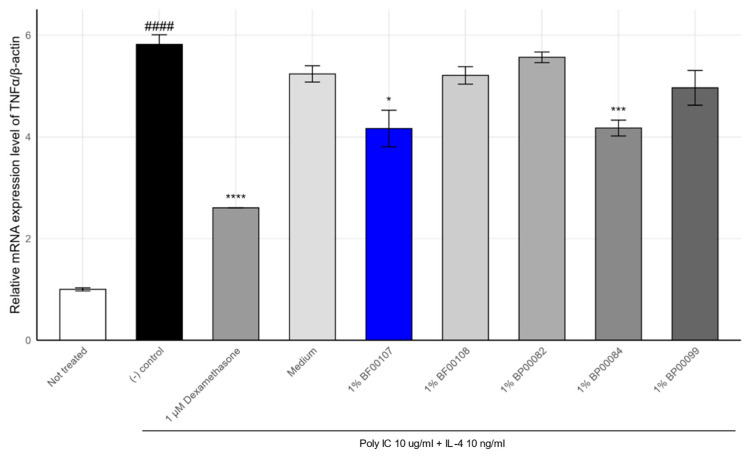
Effects of *Rothia* spp. on the mRNA expression levels of TNF-α in HaCaT cells. #### *p* < 0.0001 vs. None, * *p* < 0.05 vs. Inducer, *** *p* < 0.001 vs. Inducer, **** *p* < 0.0001 vs. Inducer.

To further evaluate the anti-inflammatory effects of *R. kristinae* BF00107, we analyzed the expression of TNF-α, a cytokine that mediates Th2-type skin inflammation, under the same inflammatory stimulation (poly I:C + IL-4) (Figure 3).

As shown in Figure 2, TNF-α expression was markedly upregulated in the stimulated control group (5.82 ± 0.19), compared to that in the untreated condition (1.00 ± 0.03, *p* = 1.45 × 10^−5^). Treatment with 1 μM dexamethasone significantly attenuated TNF-α expression to 2.60 ± 0.005 (*p* = 6.82 × 10^−5^), confirming the responsiveness of the system.

Among the tested fermentation filtrates, BF00107 showed a notable reduction i TNF-α expression (4.16 ± 0.36, *p* = 0.015), whereas other *Rothia* strains such as BF00108 (5.21 ± 0.17), BP00082 (5.57 ± 0.11), and BP00099 (4.97 ± 0.34) failed to show statistically significant suppression (*p* > 0.05). BP00084 exhibited a modest but significant reduction (4.17 ± 0.16, *p* = 0.0025), although its effect was weaker than that of BF00107.

These findings confirm the strain-dependent nature of anti-inflammatory effects within the *Rothia* genus, with *R. kristinae* BF00107 demonstrating the most consistent suppression of both IL-1β and TNF-α. Together, IL-1β and TNF-α expression data demonstrate that BF00107 selectively modulates pro-inflammatory cytokine expression in keratinocytes, suggesting its potential as a strain-specific postbiotic candidate for skin inflammation control.

### 2.3. Anti-Photoaging Effects of R. kristinae

**Figure 4:**
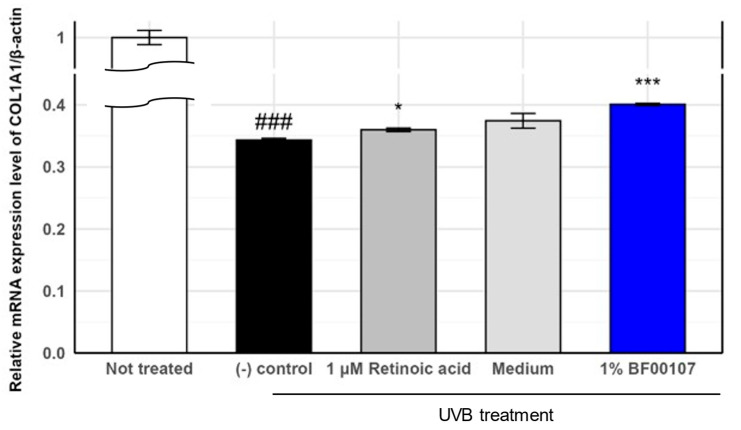
Effects of *R. kristinae* on the mRNA expression levels of COL1A1 in HaCaT cells. ### *p* < 0.001 vs. None, * *p* < 0.05 vs. UVB-treated, *** *p* < 0.001 vs. UVB-treated.

To evaluate the protective effects of *R. kristinae* BF00107 against UVB-induced damage, we measured the mRNA expression of COL1A1, a key extracellular matrix gene, in UVB-treated human fibroblasts (Figure 4). As expected, UVB irradiation significantly reduced *COL1A1* expression compared to that in the untreated control group (0.343 ± 0.00276 vs. 1.000 ± 0.0115, *p* = 6.37 × 10^−7^), confirming the detrimental impact of UVB on collagen synthesis. Treatment with 1 μM retinoic acid—a known positive control—partially restored COL1A1 expression to 0.360 ± 0.0028 (*p* = 0.0136 vs. (-) control). Furthermore, co-treatment with 1% BF00107 led to a significant recovery of COL1A1 expression to 0.401 ± 0.0016 (*p* = 5.59 × 10^−5^ vs. (-) control), indicating that the fermentation filtrate of *R. kristinae* mitigates UVB-induced suppression of collagen gene expression. Although the level of recovery was not equivalent to that of the untreated group, BF00107 demonstrated comparable or greater efficacy than retinoic acid in restoring collagen gene expression under oxidative stress.

**Figure 5:**
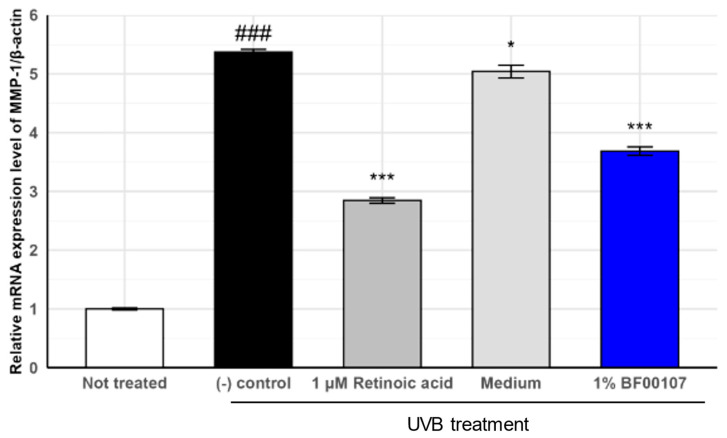
Effects of *R. kristinae* on the mRNA expression levels of MMP-1 in HaCaT cells. ### *p* < 0.001 vs. None, * *p* < 0.05 vs. UVB-treated, *** *p* < 0.01 vs. UVB-treated.

We further assessed the expression of MMP-1, a collagen-degrading matrix metalloproteinase, to further evaluate the protective effects of *R. kristinae* BF00107 against UVB-induced oxidative stress (Figure 5). UVB irradiation markedly increased MMP-1 expression to 5.372 ± 0.045, compared to that in the untreated control (1.000 ± 0.0179, *p* = 8.78 × 10^−8^), indicating severe degradation of extracellular matrix components. Treatment with 1 μM retinoic acid significantly reduced MMP-1 levels to 2.846 ± 0.0466 (*p* = 2.55 × 10^−6^ vs. (-) control), confirming its anti-photoaging effects.

Similarly, 1% BF00107 treatment significantly reduced MMP-1 expression levels to 3.686 ± 0.0699 (*p* = 3.46 × 10^−5^ vs. (-) control). Although the suppression was slightly less pronounced than that of retinoic acid, BF00107 effectively attenuated UVB-induced upregulation of MMP-1 expression, suggesting its role in the preservation of collagen integrity. Together with the COL1A1 expression data, these results suggest that *R. kristinae* BF00107 exerts a dual effect by promoting collagen synthesis and suppressing collagen degradation under UV-induced stress.

### 2.4. Transcriptomic Analysis of Rothia Species

**Figure 6:**
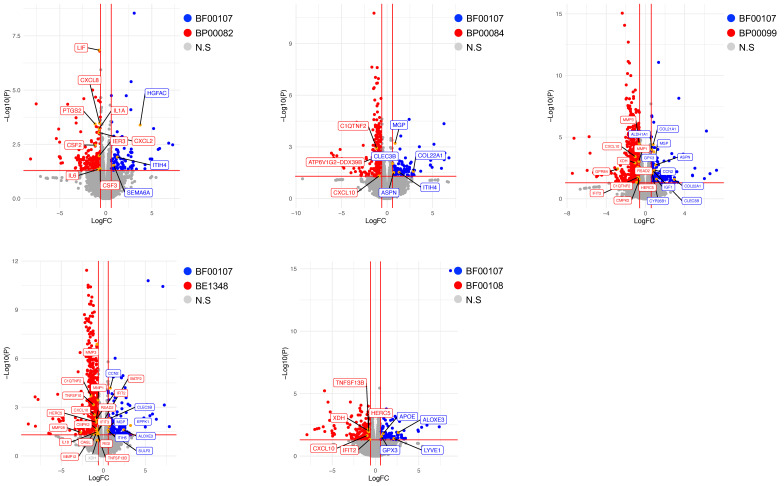
Volcano plots showing differentially expressed genes (DEGs) of fibroblasts. Vertical and horizontal red lines indicate |fold change| (FC) ≥ 1.5 and *p* < 0.05, respectively. Genes with upregulated expression in fibroblasts treated with BF00107 are indicated with blue dots. Red dots represent genes with upregulated expression in fibroblasts treated with the *Rothia* species. Statistically nonsignificant genes are indicated by gray dots.

To assess the effect of *Rothia* species on fibroblasts, we analyzed the expression of genes related to skin conditions through analysis of differentially expressed genes (DEGs). Consistent with the results of previous in vitro cell experiments, the expression of inflammatory genes such as CXCL10, IFIT2, IL-6, and IL-1α was lower in fibroblasts treated with BF00107 than in other *Rothia* species (Figure 6). Moreover, the expression of genes related to skin barrier improvement, such as APOE, CCN2, and COL22A1, was upregulated in fibroblasts treated with BF100107 (Figure 6).

### 2.5. Comparative Genomic and Transcriptomic Analyses

**Figure 7:**
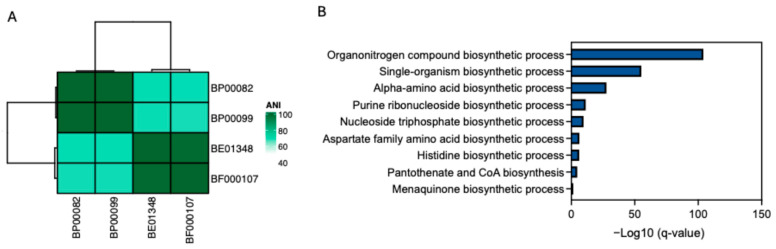
Genomic characteristics of *Rothia* species. (**A**) Average nucleotide identity (ANI) shows genomic similarity among four *Rothia* species; (**B**) Biological pathways that are associated with genes observed in two *R. kristinae* strains (BE01348 and BF00017). Only biosynthetic processes are shown.

We analyzed the genomic components of four *Rothia* species (*R. endophytica* (BP00082)*, R. amarae* (BP00099), *R. kristinae* (BE01348), and *R. kristinae* (BF00107)) to investigate the biological pathways contributing to their antiaging effects. The two *R. kristinae* strains were genomically similar to other *Rothia* species. Furthermore, we selected core genes shared by the two *R. krisinae* strains and analyzed the biological pathways of these genes. Several biosynthetic processes for organonitrogen compounds, including single amino acids, histidine, pantothenate, and CoA, are associated with these genes. Furthermore, we assessed pantothenic acid (PA), which has the potential for skin improvement as a cosmetic ingredient and has an antiaging effect on fibroblasts (Figure 7).

### 2.6. Cell Viability of PA

To evaluate the cytotoxicity of PA, cell viability was measured after treatment with various concentrations of PA (Figure S1). The relative viability values were 100.0000 ± 12.5552, 102.4600 ± 9.2706, 107.6600 ± 7.0755, and 108.3200 ± 16.9811, showing no statistically significant difference compared with the untreated control (*p* > 0.05). These results indicate that PA did not affect cell viability within the tested concentration range, confirming its non-cytotoxic nature.

### 2.7. Anti-Inflammatory Effect of PA

**Figure 8:**
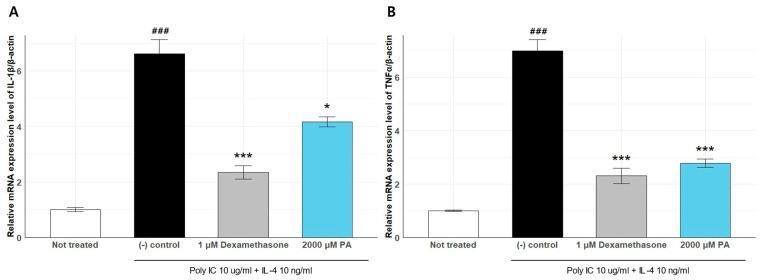
Effects of *Rothia* species on the mRNA expression levels of IL-1β (**A**) and TNF-α (**B**) in HaCaT cells. ### *p* < 0.005 vs. None, * *p* < 0.05 vs. Inducer, *** *p* < 0.001 vs. Inducer.

We quantified the expression of IL-1β and TNF-α in poly I:C + IL-4–stimulated keratinocytes to evaluate the anti-inflammatory properties of PA (Figure 8). Inflammatory stimulation significantly increased IL-1β expression to 1.878 ± 0.0215 compared to that in the untreated group (1.005 ± 0.0699), confirming the successful induction of inflammation. Treatment with 1 μM dexamethasone, a known anti-inflammatory agent, suppressed IL-1β expression to 2.345 ± 0.2422. PA treatment significantly reduced IL-1β expression (4.169 ± 0.1850 vs. negative control), indicating its strong anti-inflammatory activity.

We further assessed the expression of TNF-α under the same conditions. TNF-α expression was markedly upregulated in the stimulated control group (6.996 ± 0.4223) compared to that in the untreated condition (1.001 ± 0.0254). Dexamethasone treatment significantly attenuated TNF-α expression (2.305 ± 0.2931), and PA treatment also led to a notable reduction (2.780 ± 0.1591).

These results demonstrate that PA effectively suppresses the induction of both IL-1β and TNF-α, confirming its role as a key effector molecule that mediates the anti-inflammatory effects of *R. kristinae* BF00107.

### 2.8. Transcriptomic Analysis of PA

**Figure 9:**
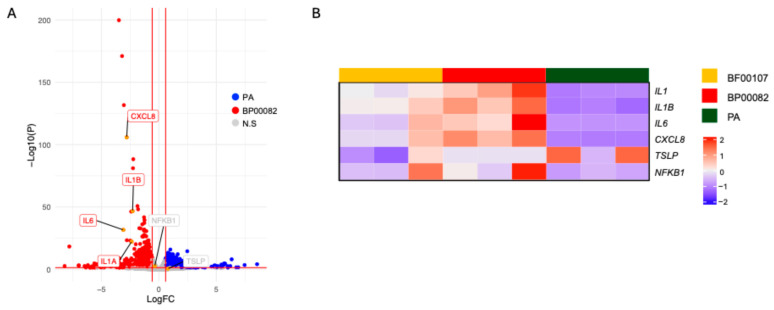
Anti-inflammatory effects of *R. kristinae* and PA. The volcano plot shows DEGs of fibroblasts. (**A**) DEGs from fibroblasts treated with BP00082 or PA. Vertical and horizontal red lines indicate |fold change| (FC) > 1.5 and *p* < 0.05, respectively; (**B**) A heatmap showing the expression patterns of inflammatory genes in fibroblasts treated with the supernatant of *R. kristinae*, *R. endophytica*, or PA. The Z-score is scaled against genes among samples.

**Figure 10:**
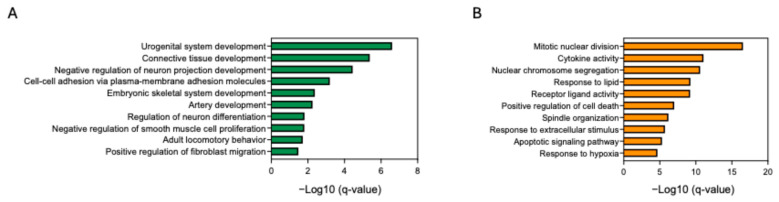
Enriched gene ontology biological processes. (**A**) Enriched processes in fibroblasts treated with PA compared to those treated with BP00082. (**B**) Enriched biological processes in fibroblasts treated with BP00082 compared to those treated with PA. Statistical significance was calculated using two-sided hypergeometric tests, and the false discovery rate was corrected using the Bonferroni step-down method.

We further assessed PA, which has the potential to improve skin as a cosmetic ingredient and has an anti-aging effect on fibroblasts. Fibroblasts were treated with PA. We then conducted whole-transcriptome sequencing; the expression of inflammation-related genes such as IL-1α, IL-1β, IL-6, CXCL8, and NFKB1 was upregulated in fibroblasts treated with BP00082 compared to fibroblasts treated with PA (Figure 9). We analyzed the enriched gene ontology (GO) of fibroblasts treated with PA and BP00082 to investigate the biological pathways that contribute to the anti-aging effect. PA treatment induced the expression of genes related to connective tissue development and positively regulated fibroblast migration. BP00082 treatment induced gene expression related to cytokine activity, positive regulation of cell death, and the apoptotic signaling pathway (Figure 10).

### 2.9. Comparative Pantothenate Production Among Rothia Species

**Figure 11:**
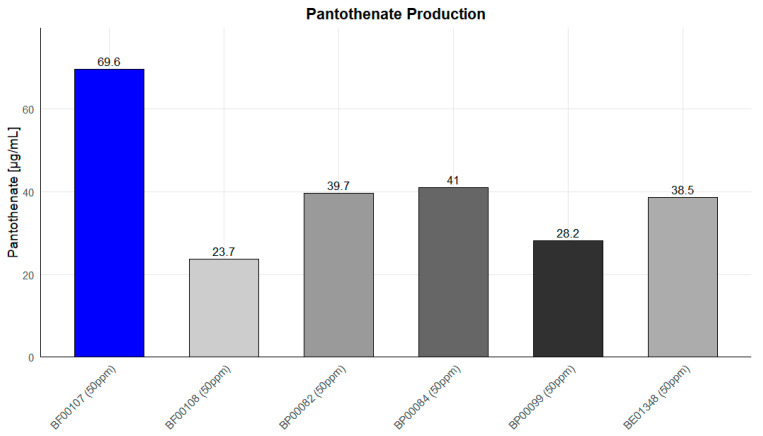
Pantothenate production levels of *Rothia* species at 50 ppm. *R. kristinae* BF00107 exhibited significantly higher pantothenate concentration than other strains.

To determine whether pantothenic acid production is a unique metabolic characteristic of *Rothia kristinae* BF00107, pantothenic acid production was compared among six *Rothia* spp. strains cultured at the same concentration (50 ppm) (Figure 11). Furthermore, to clearly identify the metabolic productivity of the strains, the pantothenic acid content of the media was measured, including SMB media as an untreated control. As a result, pantothenic acid was not detected in SMB media, confirming that the media components themselves had virtually no effect on pantothenic acid accumulation (Figure S2). Therefore, it was confirmed that the observed enhancement of pantothenic acid was entirely due to the metabolic activity of the strain. As shown in Figure 10, BF00107 showed the highest pantothenic acid accumulation at 69.6 μg/mL, which is approximately 1.7–2.9 times higher than the production (approximately 23.7–41 μg/mL) of the other five strains used as controls (BF00108, BP00082, BP00084, BP00099, and BE01348). In particular, the fact that only BF00107 showed significantly higher pantothenic acid production than other strains of the same genus under the same culture conditions suggests that this strain has a more efficiently maintained pantothenic acid biosynthetic pathway or that the related metabolic flux is preferentially regulated toward pantothenic acid synthesis. In conclusion, these results strongly demonstrate that BF00107 is a strain specialized in pantothenic acid synthesis compared to other *Rothia* strains and that it is an important candidate for the development of pantothenic acid-based functional materials or for strain-optimization research.

## 3. Discussion

This study provides the first functional validation of an *R. kristinae* strain, specifically BF00107, as a postbiotic candidate for dermatological applications. Our integrated analyses demonstrated that BF00107 exerts anti-inflammatory and anti-photoaging activities while supporting skin barrier function. These findings expand the functional repertoire of the skin microbiome beyond well-studied genera such as *Cutibacterium* and *Staphylococcus*.

At the cellular level, BF00107 markedly suppressed the induction of pro-inflammatory cytokines (IL-1β and TNF-α) in keratinocytes under poly I:C + IL-4 stimulation. The study focused on IL-1β and TNF-α because they are central mediators in keratinocyte-dependent inflammatory cascades [13,14,15]. Importantly, this effect was strain-dependent within the *Rothia* genus, as other tested species (e.g., *R. mucilaginosa* BF00108 and *R. endophytica* BP00082) failed to elicit comparable suppression. This result underscores the importance of strain-level resolution when selecting postbiotic candidates, and supports the emerging concept that minor commensals can exert distinct immunological functions.

BF00107 treatment restored COL1A1 expression and attenuated MMP-1 upregulation in fibroblasts exposed to UVB irradiation, suggesting a dual action: enhancement of collagen synthesis and prevention of extracellular matrix degradation. This opposing regulation of COL1A1 and MMP-1 reflects the fundamental imbalance between collagen synthesis and degradation that characterizes UV-induced dermal aging, and the ability of BF00107 to modulate both pathways suggests a coordinated protective effect on extracellular matrix integrity [16,17,18]. These results indicate that BF00107 preserves dermal structural integrity under oxidative stress, with an efficacy comparable to that of retinoic acid, the gold-standard anti-photoaging agent. Thus, BF00107 is a potential alternative in cosmeceutical applications.

Transcriptomic profiling provided molecular evidence for these phenotypes. As shown in Figure 5, the expression of inflammatory mediators (CXCL10, IFIT2, IL-6, and IL-1α) was downregulated in BF00107-treated fibroblasts, whereas that of barrier- and matrix-associated genes (APOE, CCN2, and COL22A1) was upregulated. CXCL10, IFIT2, IL-6, and IL-1α are well-established mediators of innate immune activation, interferon signaling, and pro-inflammatory responses, as demonstrated across multiple studies [19,20,21,22]. The coordinated upregulation of these genes in our system is consistent with previous reports showing their induction following pathogen-associated molecular pattern (PAMP) stimulation and interferon pathway activation. APOE, CCN2, and COL22A1 are key regulators of extracellular matrix organization, tissue repair, and microenvironmental homeostasis, as previously characterized in studies focusing on tissue remodeling and structural integrity [23,24,25]. The expression changes observed in our analysis reflect their established involvement in maintaining or reconfiguring tissue architecture under stress or injury. This coordinated response demonstrates that BF00107 not only suppresses inflammation but also reinforces structural and barrier-related pathways. These signatures were absent in the fibroblasts treated with other *Rothia* species, further confirming the strain specificity of BF00107.

Genomic and metabolomic analyses revealed that BF00107 harbors a complete pantothenate biosynthesis pathway and produces significantly higher levels of pantothenic acid (vitamin B5) than other *Rothia* strains. Functional assays with purified pantothenate demonstrated suppression of IL-1β and TNF-α, recapitulating the effects of BF00107. Moreover, transcriptomic analysis showed that pantothenate treatment induced gene expression patterns overlapping with those of BF00107, including the suppression of inflammatory genes and the induction of connective tissue development pathways (Figure 7 and Figure 8). These findings strongly suggest that pantothenate is the principal effector metabolite that mediates the dermatological benefits of BF00107.

In addition to its functional efficacy, BF00107 passed all safety assessments, including the human repeat insult patch test (HRIPT), Ames test, and in vitro irritation tests on the skin and eyes. These results further support the potential for its safe incorporation into dermocosmetic formulations.

Despite these promising findings, this study had several limitations. First, although pantothenate has emerged as a key mediator, the causality has not been definitively established. Targeted inhibition and neutralization studies are required to validate their direct roles in each phenotype. Second, cytokine regulation was evaluated only at the mRNA level, and direct protein quantification (e.g., by immunochemical assays) was not performed. Therefore, future studies employing ELISA or Western blot analyses are necessary to verify whether transcriptional suppression correlates with actual protein-level changes. Third, the study was limited to in vitro models; in vivo and clinical investigations with diverse populations are essential to confirm efficacy and safety under physiological conditions. It should also be noted that keratinocyte- and fibroblast-based in vitro assays capture only a limited portion of the complex immunological interactions present in living skin. Because cytokine signaling in vivo involves multiple immune cell subsets, barrier–immune crosstalk, and systemic regulatory pathways, the transcriptional changes observed in our simplified models cannot be directly extrapolated to in vivo immune environments. Therefore, the immunomodulatory effects identified here require validation through in vivo or clinical studies to establish their physiological relevance. Finally, BF00107 likely produces additional bioactive metabolites that were not explored in this study. Therefore, comprehensive metabolomic and proteomic profiling is required to fully characterize its functional repertoire.

In conclusion, BF00107 expands the scope of skin microbiome-derived postbiotics by combining strong anti-inflammatory and photoaging-protective effects with unique pantothenate-producing capacity. Its strain-specific activity, together with confirmed safety, highlights the untapped potential of rare commensals as bioactive reservoirs. These findings not only provide a mechanistic rationale for incorporating BF00107 into dermocosmetic formulations but also underscore the broader value of mining underexplored skin taxa for novel functional ingredients.

## 4. Materials and Methods

### 4.1. Microbial Isolation and Culture

To develop skin-beneficial microbiome ingredients, microorganisms in the genus *Rothia* (Table 1; including *R. kristinae* BF00107 (Multirenol™); accession number: KCCM12685P), were isolated from facial skin samples as suggested by an algorithm based on a previous study [4,26]. The collection of human facial skin samples was approved by the Institutional Review Board (IRB Protocol Number: HBABN01-210217-HR-0181-01) of the H&BIO Corporation R&D CENTER (H&BIO, Seongnam, Republic of Korea). The isolated bacteria were stored at the Korean Culture Center of Microorganisms (Seoul, Republic of Korea) and COSMAX Microbiome Bank (COSMAXBTI Inc., Seongnam, Republic of Korea). A single colony of each bacterium was selected from the plate and grown overnight in a skin-mimetic broth (SMB). The SMB consisted of 1.06 g glucose, 0.2 g rhamnose, 0.013 g fructose, 0.05 g MgSO4, 2.5 g K_2_HPO_4_, 5 g NaCl, and 1 g of yeast extract in a liter of distilled water. The overnight culture was then diluted 1:100 in fresh SMB medium (50 mL), transferred into a 250 mL Erlenmeyer flask, and incubated at 30 °C on a shaker (160 rpm) for 48 h in the dark. After incubation, SMB medium (control) and the culture at 1 × 10^8^ CFU/mL centrifuged at 8000 rpm for 10 min. They were filtered using a syringe filter (0.45-μm pore size; Minisart, Sartorius, Germany). All culture filtrates were prepared in triplicates [27].

### 4.2. Cell Line and Cell Culture (Human Keratinocyte HaCaT)

The HaCaT human keratinocyte cell line was obtained from the American Type Culture Collection (ATCC; Manassas, VA, USA) and maintained in Dulbecco’s modified Eagle’s medium (DMEM; HyClone, Logan, UT, USA) supplemented with 10% fetal bovine serum (FBS) and 1% antibiotic–antimycotic solution. Cells were cultured at 37 °C in a humidified incubator with 5% CO_2_ and used for experiments at approximately 80% confluence. Before some treatments, HaCaT cells were pre-incubated with menthol (100 μM) in serum-free medium for 24 h to induce a controlled stress response.

#### 4.2.1. Cell Viability Assay (MTT Assay)

HaCaT cells were exposed to various concentrations of *R. kristinae* (0.01, 0.1, and 1%) in serum-free Dulbecco’s modified Eagle’s medium (DMEM) for 24 h. Cell viability was assessed by adding 20 μL of 3-(4,5-dimethyl-2-thiazolyl)-2,5-diphenyl-2H-tetrazolium bromide (MTT) solution (5000 μg/mL) to each well, and the plates were incubated for 4 h. Following incubation, the supernatant was removed, the formazan crystals were dissolved in 100 μL of dimethyl sulfoxide (DMSO), and cell viability was assessed by comparing the absorbance measured at 540 nm using a microplate reader relative to the untreated controls.

#### 4.2.2. Inflammatory Model and Treatment

For the inflammatory response model, the HaCaT cells were stimulated with 10 μg/mL of polyinosinic–polycytidylic acid (poly I:C) and 10 ng/mL of IL-4 in the presence of 1% antibiotic–antimycotic to mimic an inflammatory environment [28]. After 24 h of stimulation, *Rothia* spp. (1%), the anti-inflammatory positive control dexamethasone (1 μM; Sigma-Aldrich, MO, USA), or PA were added to the cells in serum-free medium and incubated for 4 h. Dexamethasone inhibits the activation of the Toll-like receptor 3 signaling pathway by poly I:C, thereby suppressing inflammation and reducing the release of pro-inflammatory cytokines [29]. Cells were then collected to analyze the expression of inflammatory genes (e.g., IL-1β and TNFα). The cytokines IL-1β and TNF-α were selected as primary readouts because they represent central mediators of keratinocyte-driven inflammatory responses and are well-established markers of TLR3- and Th2-associated skin inflammation relevant to the poly I:C and IL-4 stimulation model [13,14,15]. IL-1β is a key pro-inflammatory cytokine produced by keratinocytes that initiates downstream cytokine cascades and amplifies cutaneous inflammatory responses, and TNF-α is a central mediator released by keratinocytes following TLR activation, driving innate immune signaling and propagating epidermal inflammation.

### 4.3. Cell Line and Cell Culture (Human Dermal Fibroblast Hs68)

The human dermal fibroblast Hs68 cell line (ATCC) was cultured in DMEM supplemented with 10% FBS and 1% antibiotic–antimycotic at 37 °C in a humidified incubator with 5% CO_2_. For experiments, cells were seeded into 6-well plates at approximately 80% confluence and incubated for 24 h before treatment, according to the experimental purpose.

UVB-Induced Photoaging Model and Treatment

For collagen type I alpha 1 chain (COL1A1) and matrix metalloproteinase-1 (MMP-1) assays under UV-irradiated conditions, Hs68 medium was removed, Dulbecco’s phosphate-buffered saline (DPBS; Gibco, Waltham, MA, USA) was added, and the cells were irradiated with or without 12 mJ/cm^2^ UVB (wavelength 290–320 nm, maximum peak 311 nm). After UVB irradiation, DPBS was removed and replaced with FBS-free DMEM. The *R. kritstinae* culture filtrate [1% (*w*/*w*)] was treated and further incubated for 24 h. A sample treated with UV light but without culture filtrate treatment was used as a negative control COL1A1 and MMP-1 were selected as representative photoaging markers because UV irradiation is known to suppress COL1A1-mediated collagen synthesis while strongly inducing MMP-1-driven collagen degradation, making these genes well-established indicators of extracellular matrix disruption under UV-induced oxidative stress [16,17,18].

### 4.4. RNA Extraction from Human Cells and qPCR Analysis

Total RNA was extracted from the cells using TRIzol reagent (Takara, Kusatsu, Japan) according to the manufacturer’s instructions. Residual RNA was further purified using the RNeasy Mini Kit (Qiagen, Hilden, Germany). Complementary DNA (cDNA) was synthesized from 1 μg of total RNA using reverse transcription premix (Elpis-Biotech, Daejeon, Republic of Korea) under the following reaction conditions: 45 °C for 45 min and 95 °C for 5 min. Quantitative real-time PCR (qPCR) was performed using a StepOne Plus™ System Software (v2.3, Applied Biosystems, Foster City, CA, USA) with SYBR Green PCR Master Mix (Applied Biosystems) according to the manufacturer’s instructions. The following gene-specific primers were used in the ABI 7300 system (Bioneer, Daejeon, Republic of Korea): β-actin (F: 5′-GGCCATCTCTTGCTCGAAGT-3′ and R: 5′-GACACCTTCAACACCCAGC-3′), IL-1β (F: 5′-GTCATTCGCTCCCACATTCT-3′ and R: 5′-ACTTCTTGCCCCCTTTGAAT-3′), TNFα (F: 5′- CTCTTCTGCCTGCTGCACTTTG-3′ and R: 5′-ATGGGCTACAGGCTTGTCACTC-3′), MMP-1 (F: 5′-CGAAATTTGCCGACAGAGATGA-3′ and R: 5′-GTCCCTGAACAGCCCAGTACTT-3′), and COL1A1 (F: 5′-CTCGAGGTGGACACCACCCT-3′ and R: 5′-CAGCTGGATGGCCACATCGG-3′). The thermal cycling conditions were set as follows: initial activation at 50 °C for 2 min and 95 °C for 10 min, followed by 40 amplification cycles at 95 °C for 10 s and 60 °C for 1 min. The expression of β-actin was used as an internal control for normalization.

### 4.5. Sequencing and Bioinformatic Analysis

#### 4.5.1. Sample Preparation for RNA and Whole-Genome Sequencing

Only human dermal fibroblasts were subjected to RNA-seq analysis in this study. No bacterial mRNA isolation or bacterial transcriptomic profiling was performed, as the sequencing component focused solely on host transcriptional responses.

For transcriptomic analysis, total RNA was extracted from Hs68 fibroblasts treated with bacterial culture filtrates of *R. kristinae* and other *Rothia* species. Cells were harvested under three experimental conditions: (1) untreated control (NC), (2) inflammation-induced control (poly I:C 10 µg/mL + IL-4 10 ng/mL), and (3) the culture filtrates of *R. kristinae* and other *Rothia* species–treated after inflammation. High-quality RNA (RIN > 7) was obtained using the QIAzol Lysis Reagent and RNeasy Mini Kit (Qiagen, Germany). DNase I treatment was applied to eliminate residual genomic DNA prior to sequencing.

For genomic analysis, the complete *R. kristinae* genome was extracted using the HiGene™ Genomic DNA Prep Kit for Microorganisms (Biofact, Daejeon, Republic of Korea) after mechanical disruption with a FastPrep-24 5G homogenizer (MP Biomedicals, Solon, OH, USA). The extracted DNA was quantified using a Qubit 4 fluorometer (Thermo Fisher Scientific, Waltham, MA, USA), and the library size distribution was assessed using a 4150 TapeStation System (Agilent, Santa Clara, CA, USA).

#### 4.5.2. Library Construction and Sequencing

For Hs68 fibroblasts, RNA-sequencing (RNA-seq) libraries were constructed using a TruSeq Stranded mRNA Sample Preparation Kit (Qiagen). In brief, poly-A mRNA was isolated from 1 μg total RNA per sample using oligo (dT) magnetic beads and then fragmented at high temperature. First-strand cDNA was synthesized using SuperScript II reverse transcriptase (Invitrogen, Carlsbad, CA, USA) and random primers, followed by second-strand cDNA synthesis using DNA Polymerase I and RNase H. Double-stranded cDNA fragments were end-repaired, A-tailed, and ligated to indexed adaptors. The libraries were PCR-amplified and their quality assessed using an Agilent TapeStation D1000 ScreenTape system. Sequencing was performed on an Illumina NovaSeq X platform (Illumina, San Diego, CA, USA) to generate 2 × 151 bp paired-end reads. For the complete *R. kristinae* genome, DNA sequencing libraries were constructed using the SMRTbell prep kit 3.0 (PacBio, Menlo Park, CA, USA). Long-read sequencing was performed using a PacBio Sequel IIe (PacBio).

#### 4.5.3. Gene Expression Analysis

Raw FASTQ files were generated using Illumina bcl2fastq with adapter trimming enabled during demultiplexing. Read quality was assessed using FastQC (v0.11.9), which confirmed high-quality reads (Q30 > 90%) and negligible adapter contamination; therefore, no additional trimming was required. Clean reads were aligned to the human reference genome (GRCh38) using STAR (v2.7.11b) in 2-pass mode with default parameters. Gene- and transcript-level abundances were quantified using RSEM (v1.3.1) [30,31]. The expected count values produced by RSEM were normalized using the TMM method implemented in edgeR (v4.4.2), and differentially expressed genes (DEGs) between BF00107, BP00082, and PA were identified using edgeR with a *p* value < 0.05 and |fold change| > 1.5 [32]. Transcripts per kilobase million (TPM) values were calculated using RSEM following STAR alignments. TPM values were log2-transformed and converted to z-scores across samples to identify gene expression patterns.

#### 4.5.4. Construction of the *Rothia* Genome

The quality of sequenced reads was evaluated using filtlong with the -min_length 1000 -keep_percent 95 settings. These were then assembled using Trycycler with the default setting. The bacterial genome was annotated using Prokka with default settings. Pangenome analysis of the four *Rothia* species was performed using Roary with default settings. The assembled genome contigs were compared using JSpeciesWS to identify the genomic similarity among the four *Rothia* species. Bacterial genes were input into the Cytoscape plug-in ClueGO v2.5.10 to annotate the functionally grouped networks. Functionally related Gene Ontology (GO) terms for biological processes and KEGG in *Escherichia coli* (version: 18 November 2016, and 23 May 2024, respectively) were grouped based on a kappa score >0.4. The *p* value cutoff for significance was 0.05, and the correction method was Bonferroni step-down. The GO term fusion was used. The minimum and maximum GO levels were 3 and 8, respectively.

### 4.6. Pantothenate Quantification Using High Performance Liquid Chromatography (HPLC)

The pantothenic acid concentration in the bacterial supernatant was quantified by HPLC (1260 Infinity II, Agilent Technologies, Santa Clara, CA, USA). After bacterial culture, SMB medium (control) and the *Rothia* spp. were collected at 1 × 10^8^ CFU/mL and centrifuged at 6000 rpm for 10 min. Only the supernatant was lyophilized using a centrifuge (5804R, Eppendorf, Hamburg, Germany). The lyophilized culture solution was dissolved in the initial mobile phase (TFA 0.025% in water) and diluted to 50 ppm. Additionally, high-molecular-weight substances, including proteins, were removed from the solution using a 0.22 μm nylon filter to facilitate analysis. The column used was InfinityLab Poroshell 120 EC-C18 (150 × 4.6 mm), and the column temperature was set to 23 °C, the injection volume to 10 μL and the flow rate to 1 mL/min. Mobile phase A used 0.025% TFA in water, and mobile phase B used acetonitrile; the ratio was changed over time (Table 2). The absorption wavelength was set to 210 nm, and the analysis was performed with a variable wavelength detector (VWD). The standard substances were measured at concentrations of 10 ppm, 20 ppm, 40 ppm, 60 ppm, 80 ppm and 100 ppm to construct a quantitative curve.

### 4.7. Statistical Analysis

All experiments were conducted at least in triplicates. The data are presented as the mean ± standard deviation. For in vitro cell assay analysis, statistical analysis was performed using the SPSS statistical package (version 25.0; IBM Corp., Armonk, NY, USA). Statistical comparisons between two groups were performed using a two-tailed Student’s *t*-test. For all tests, *p*-value < 0.05 was considered statistically significant, with *p*-value < 0.01 denoted as highly significant.

### 4.8. Safety Assessments

Safety evaluations for *R. kristinae* BF00107 culture filtrate were performed using a human repeat insult patch test (HRIPT) and three in vitro toxicity tests: the bacterial reverse mutation (Ames) test, an in vitro skin irritation test, and an in vitro eye irritation test (Dermapro, Seoul, Republic of Korea; Biotoxtech, Cheongju, Republic of Korea).

The HRIPT was conducted to assess the primary irritation and sensitization potential of *R. kristinae* BF00107 culture filtrate. Fifty-two healthy volunteers (1 male and 51 females; mean age, 50.04 ± 7.84 years) participated in the study. Test patches (8 mm Finn Chamber^®^) were applied on the volar forearm. During the induction phase, the test material was applied nine times over three weeks (three times per week) under occlusion for 24 h. Skin reactions were evaluated 24 or 48 h after patch removal. After a two-week resting phase, the challenge phase was performed by applying the same sample to a naïve site for 48 h, and skin responses were assessed at 20 min, 24 h, and 48 h after patch removal.

The in vitro skin irritation test was performed using the reconstructed human epidermis (RhE) model according to OECD Test Guideline (TG) 439, “In Vitro Skin Irritation: Reconstructed Human Epidermis Test Method.” The in vitro eye irritation test was conducted using the reconstructed human cornea-like epithelium (RhCE) model following OECD TG 492, “Reconstructed Human Cornea-like Epithelium (RhCE) Test Method for Identifying Chemicals Not Requiring Classification for Eye Irritation or Serious Eye Damage”.

Additionally, the bacterial reverse mutation test (Ames test) was carried out following OECD TG 471, using *Salmonella typhimurium* (TA98, TA100, TA1535, TA1537) and *Escherichia coli* (WP2uvrA) strains, both in the presence (+S9) and absence (−S9) of metabolic activation.

All toxicity tests were conducted in compliance with the Organisation for Economic Co-operation and Development (OECD) guidelines: TG 439 (Skin Irritation: Reconstructed Human Epidermis Test Method), TG 492 (Eye Irritation: RhCE Model), and TG 471 (Bacterial Reverse Mutation Test), under Good Laboratory Practice (GLP) conditions.

## 5. Conclusions

This study provides the first comprehensive evaluation of the dermatological potential of *R. kristinae* BF00107, a skin-derived commensal strain with unique functional properties. In vitro models, comparative genomics, and metabolite profiling revealed that the fermentation filtrate of *R. kristinae* BF00107 exerts notable anti-inflammatory and anti-photoaging effects, improves skin barrier integrity, and enhances overall skin hydration.

Mechanistically, these effects are likely mediated in part by the pantothenate biosynthetic capacity of the strain, which may contribute to both the structural and immunological modulation of skin tissue. Importantly, the observed benefits were strain specific within the *Rothia* genus, highlighting the necessity of fine taxonomic resolution in microbiome-based ingredient discovery.

Our findings suggest that *R. kristinae* BF00107 is a promising postbiotic candidate for future dermocosmetic applications, particularly in formulations targeting inflammation, photoaging, and barrier repair. Further studies exploring the full spectrum of secreted metabolites and their long-term clinical efficacy is required to advance the therapeutic development of this strain.

## Figures and Tables

**Table 1:** List of *Rothia* and related strains used for functional and genomic evaluation.

No	Species Name	Strain Name
1	*Rothia kristinae*	BF00107
2	*Rothia mucilaginosa*	BF00108
3	*Rothia endophytica*	BP00082
4	*Rothia endophytica*	BP00084
5	*Rothia amarae*	BP00099
6	*Rothia kristinae*	BE01348

**Table 2:** Gradient Profile for the HPLC Method.

Time (min)	Solvent A (%)	Solvent B (%)
0	100	0
5	100	0
11	75	25
19	55	45
20	60	40
22	100	0

## Data Availability

The original contributions presented in this study are included in the article. Further inquiries can be directed to the corresponding authors.

## Supplementary Materials

The following supporting information can be downloaded at: https://www.mdpi.com/article/10.3390/ijms262412058/s1.

## Abbreviations

The following abbreviations are used in this manuscript:

APOE	Apolipoprotein E
CCN2	Cellular Communication Network Factor 2
COL1A1	Collagen type I alpha 1
COL22A1	Collagen Type XXII Alpha 1 Chain
CXCL10	C-X-C Motif Chemokine Ligand 10
DMEM	Dulbecco’s modified Eagle’s medium
DMSO	Dimethyl sulfoxide
FBS	Fetal bovine serum
IFIT2	Interferon-Induced Protein with Tetratricopeptide Repeats 2
IL-1α	Interleukin-1 alpha
IL-1β	Interleukin-1 beta
IL-6	Interleukin-6
IL-8	Interleukin-8
MMP-1	Matrix metalloproteinase-1
NF-κB	Nuclear factor kappa-light-chain-enhancer of activated B cells
PA	D-Pantothenic acid hemicalcium salt
poly I:C	Polyinosinic–polycytidylic acid
RA	Retinoic acid
TNFα	Tumor necrosis factor-α
UVB	Ultraviolet B
