# Supplementary material for: Functional Expansion of the Skin Microbiome: A Pantothenate-Producing Rothia Strain Confers Anti-Inflammatory and Photoaging-Protective Effects
Source: Int J Mol Sci. 2025 Dec 15;26(24):12058. doi: 10.3390/ijms262412058 (PMC12732660; doi:10.3390/ijms262412058)
Supplement: Supplementary file 1 [file ijms-26-12058-s001.zip › ijms-3953660-supplementary.pdf]

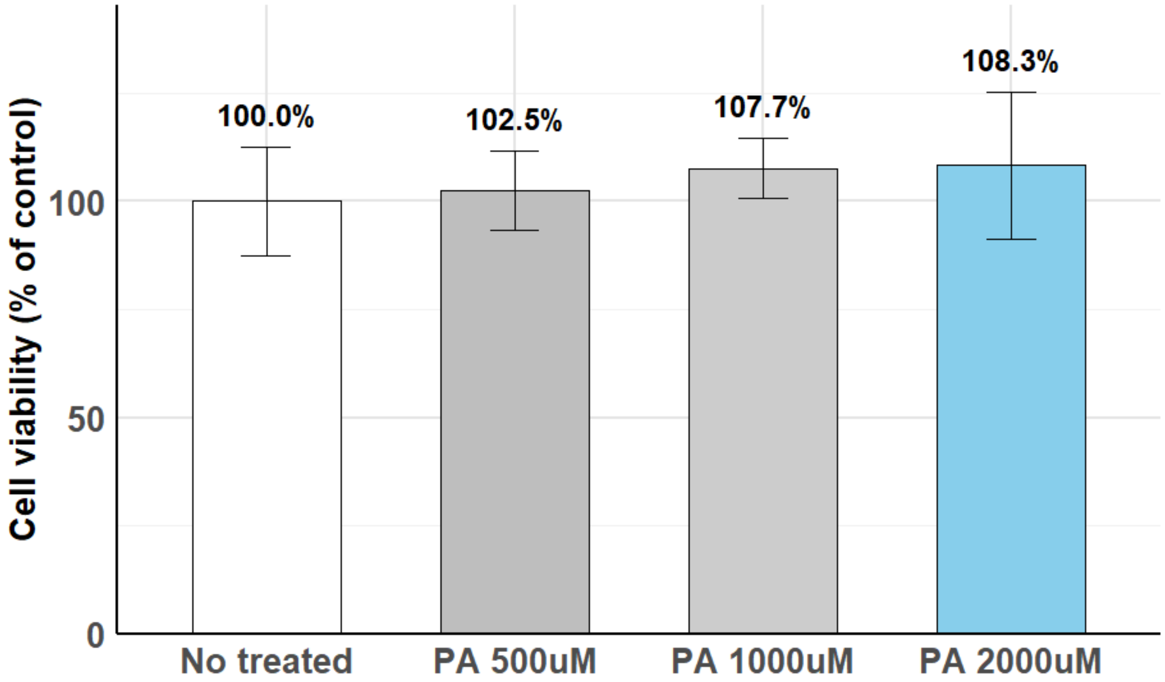

Figure S1. Effects of pantothenic acid on cell viability. Cells were treated with pantothenic acid (500–2000μM) for 24 h. Pantothenic acid at 500–2000μM slightly increased cell viability compared with the none but showed no significant effects. Data are shown as mean ± SD (n = 4); statistical significance was assessed using a two-tailed Student's *t*-test.

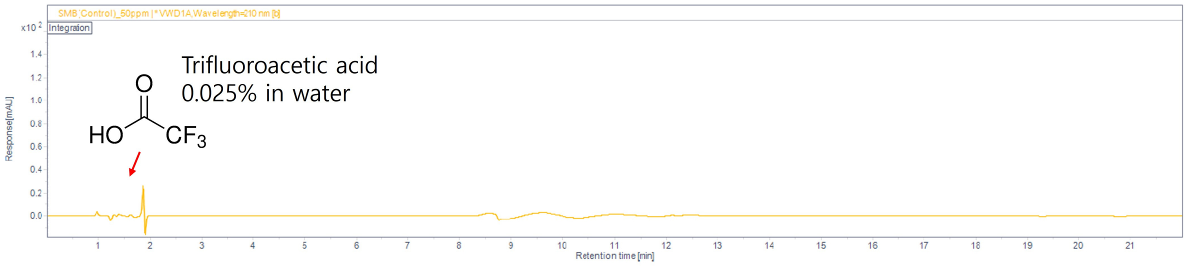

Figure S2. Pantothenic acid analysis in SMB medium. No pantothenic acid was detected, indicating negligible contribution of medium components to its accumulation.
